# Virus-Like Attachment Sites and Plastic CpG Islands: Landmarks of Diversity in Plant Del Retrotransposons

**DOI:** 10.1371/journal.pone.0097099

**Published:** 2014-05-21

**Authors:** Guilherme M. Q. Cruz, Cushla J. Metcalfe, Nathalia de Setta, Edgar A. O. Cruz, Andréia Prata Vieira, Rosario Medina, Marie-Anne Van Sluys

**Affiliations:** 1 Departamento de Botânica, Instituto de Biociências (IB), Universidade de São Paulo (USP), São Paulo, São Paulo, Brasil; 2 Universidade Federal do ABC (UFABC), São André, São Paulo, Brasil; Georgia Institute of Technology, United States of America

## Abstract

Full-length Del elements from ten angiosperm genomes, 5 monocot and 5 dicot, were retrieved and putative attachment (*att*) sites were identified. In the 2432 Del elements, two types of U5 *att* sites and a single conserved type of U3 *att* site were identified. Retroviral *att* sites confer specificity to the integration process, different *att* sites types therefore implies lineage specificity. While some features are common to all Del elements, CpG island patterns within the LTRs were particular to lineage specific clusters. All eudicot copies grouped into one single clade while the monocots harbour a more diverse collection of elements. Furthermore, full-length Del elements and truncated copies were unevenly distributed amongst chromosomes. Elements of Del lineage are organized in plants into three clusters and each cluster is composed of elements with distinct LTR features. Our results suggest that the Del lineage efficiently amplified in the monocots and that one branch is probably a newly emerging sub-lineage. Finally, sequences in all groups are under purifying selection. These results show the LTR region is dynamic and important in the evolution of LTR-retrotransposons, we speculate that it is a trigger for retrotransposon diversification.

## Introduction

With a very few exceptions, transposable elements (TEs) are ubiquitous in eukaryotic genomes. Most copies of TEs in a genome are either defective, fossilized or are restrained by host silencing mechanisms. Despite this they can reach high copy numbers and become the major component of a genome. Long terminal repeat retrotransposons (LTR-RTs) are the predominant order of TEs found in plant genomes [1], 75% of the maize genome and 54% of the sorghum genome are LTR-RTs [2]. The long terminal repeats (LTRs) at the 5′ and 3′ ends of the element contain the regulatory elements of the LTR-RT, such as the promoters, enhancers and termination signals. LTRs can also act as novel promoters or enhancers to neighboring cellular genes, driving changes in expression patterns [3].

The coding domains of TEs are relatively well conserved over genera and kingdoms and are used for phylogenetic analyses [4]. Among the non-coding domains, the LTR region is the most variable [5], [6] and is structurally divided into three well-defined regions: the U3, R and U5. The promoter and other regulatory elements are located within the U3 [7]. Transposable elements are often kept in a silenced state in plants by promoter targeted methylation [8]. Since the LTR-RT promoter is located within the U3 region, a known target of several small RNAs [9], [10], it is probably a key region for host control.

Retrotransposons containing LTRs are mobile DNA elements that replicate via RNA intermediates. In their structure and mobility they resemble retroviruses, except that they are unable to move from cell to cell [11]. In both retroviruses and LTR-RTs, integration is mediated by an integrase protein. Two conserved motifs, one at the 5′ end of the U3 region and the other at the 3′ end of U5 region, called attachment (*att)* sites, have been identified in retroviruses. Recognition of *att* sites by the retroviral integrase confers specificity to the integration process [12]–[14]. Despite the structural similarity of LTR-RTs to retroviruses, *att* sites have only been briefly described for LTR-RTs [15], [16].

Ty3/Gypsy and Ty1/Copia are the two most represented superfamilies in plant genomes and are abundant in both monocot and eudicot genomes [17], [18]. Within these superfamilies, evolutionary lineages have been identified which have distinct patterns in terms of structure, expression, regulation and chromosomal distribution [10], [17]. Del, a Ty3/Gypsy lineage, has the largest described LTRs, and also the largest LTR length variation, from 1.1 to 4.4 kb [18]. Elements from this lineage (also described as Tekay) are found in all plant genomes examined, under various names, such as *Retrosat-2* in *Oryza sativa*, *Tma* and *Legolas* in *Arabidopsis thaliana* and *Peabody* in *Pisum sativum* [18]. The scope of the present work was to explore in more depth the variability in length, sequence and structure of the LTRs and *att* sites.

Most of the knowledge gained in terms of TE structure, regulation and fate has been derived from model organisms, but with the release of several plant genomes in the last 10 years it is now possible to address new questions. With no a priori information available, we used a structural and Hidden Markov Model (HMM) based approach to extract and classify full-length LTR-retrotransposons from 10 sequenced angiosperm genomes. The Del linage elements were classified into groups based on a phylogenetic analysis of coding domains. The structure of the LTR between and within groups was examined. We identified putative *att* sites in LTR-RTs, with two sequence variants found in the U5 *att* and a U3 *att* conserved among all studied genomes. We report that CpG islands are often found within the LTR, in some groups there is a 5′ CpG island which is highly variable in length and sequence when compared to the rest of the LTR. To advance our understanding of the dynamics of Del elements we also examined the distribution of LTR-RTs within chromosomes in sorghum and maize sequenced genomes and tested which type of selective constraint all Del groups are evolving under. Del elements are unevenly distributed among chromosomes, a pattern not previously reported. All groups are evolving mainly under purifying selection, which we suggest represents a high selective constraint due to the transposition process.

## Materials and Methods

### Del Element Extraction and Classification

Ten fully sequenced genomes (*Aradopsis thaliana, Brachypodium distachyon, Glycine max, Medicago truncatula, Populus trichocarpa, Oryza sativa, Setaria italica, Sorghum bicolor, Vitis vinifera* and *Zea mays*,) were downloaded (11/25/2011) from the plantGDB ftp website (ftp://.ftp.plantgdb.org/download/Genomes). The complete genome sequences were split into sequences from individual chromosomes and screened using LTR_STRUC [19] with default parameters. HMM profiles were built using the HMMER package (version 2.3.2) based on reverse transcriptase (RT) amino acid alignments previously described [10]. Extracted sequences were conceptually translated in all six frames and subjected to a HMMscan (HMMER 2.3.2 package) against the HMM profiles, with a e-value cut-off of 1e^−10^. All sequences were classified into lineages [17], [18] according to the best match. Further analyses were done just on elements classified as being from the Del lineage, 2432 sequences. All sequences and alignments are available on request.

### Phylogenetic Analysis

Del sequences were assigned to groups by phylogenetic analysis. Two phylogenies were inferred, the first based on the RT and part of the RNaseH coding domains, the second on the integrase domain. The RT-RNaseH and integrase domains were excised, aligned using k-align or Muscle [20] and adjusted manually. The optimal model of nucleotide substitution was estimated using MEGA5 [21] with default settings. A neighbor-joining phylogeny was inferred with MEGA5 using the highest-ranked substitution model available (Tamura 3-parameter) and a bootstrap of 100 replicates. Sequences from the Reina, CRM and Galadriel families [10], [18] were used as outgroups. Nine well-supported major branches were identified and named groups I to IX. Branches within each group were called Subgroups a, b, etc.

### Identifying Putative Attachment (*att*) Sites

Two conserved regions were identified, one at the 5′ end of the LTR, and a second, different region at the 3′ end of the LTR, by examining alignments of all Del sequences in Jalview (version 2.4.0.b2) using the option “color *per* conserved sites” [22]. The regions were identified as putative *att* sites and called the U3 *att* (at the 5′ end of the LTR) and the U5 *att* (at the 3′ end of the LTR). The conserved ten base pair U3 *att* was identified by examining the first forty base pairs in an unaligned multifasta file. The second, eleven base pair U5 *att* was identified by reverse-complementing extracted LTR sequences. The first and last 40 bases of the LTRs were submitted to WebLogo (http://weblogo.berkeley.edu) to examine the sequence conservation of the *att* sites. U5 *att* sequence was sorted according to similarity in Jalview [22] and split into two groups. The U3 *att* sequence was split according to genome. All sets of *att* sequences were plotted using WebLogo. In order to quantify conservation of the 5′ and 3′ regions, the overall mean p-distance was calculated using the program MEGA 5 [21].

### Exploring Differences in Del LTRs

LTR lengths were taken from the LTR_STRUC output. For each subgroup identified by the phylogenetic analysis, the average LTR length and the standard deviation was calculated and plotted in Microscoft Excel. Outliers in the graph for the maize genome were examined further. Using a self-BLASTn to find the ends of the elements we manually examined 50 outliers and found that the LTRs had been incorrectly extracted by LTR_STRUC. For further analyses involving LTR length the data was removed if the LTR length was three standard deviations above or below the average for each subgroup.

The length of the LTR and the complete element were taken from the LTR_STRUC output. The length of the internal coding region for each element was calculated by subtracting two times the length of the LTR from the length of the complete element. The length of the LTR was plotted against the length of the complete element and against the length of the internal coding region in Microsoft Excel. The length of the complete element was also plotted against the length of the internal coding region in Microsoft Excel.

LTR alignments for each subgroup were used to identify and characterize CpG islands. If the subgroup included more than one type of LTR, they were split and analyzed separately. The consensus sequence of each alignment was submitted to the EMBOSS CpG island tool (http://www.ebi.ac.uk/Tools/emboss/cpgplot/) to identify any islands and to calculate %GC content. To compare CpG islands identified with regions of sequence variance/conservation, each alignment was also submitted to the EMBOSS conservation plot tool (http://emboss.bioinformatics.nl/cgi-bin/emboss/plotcon). The outputs showing sequence conservation and %GC content were manually overlapped with the coordinates of any CpG islands identified.

To determine if the 5′ CpG island was more variable in length, which is more ‘plastic’, than the rest of the LTR we calculated a “plasticity ratio” for each subgroup. For each subgroup, gaps in the alignments were removed and the mean length and standard deviation were calculated, using Microsoft Excel, for the CpG island and the rest of the LTR. The standard deviation was divided by the mean length to give a “plasticity ratio”.

### Distribution of Del among Chromosomes

The distribution of full-length Del elements and U3 *att* matches were examined in the two genomes (sorghum and maize) with the highest copy number of elements, according to the LTR_STRUC output. To map full-length copies, data was taken from the LTR_STRUC output, which included the source of the LTR-RT, in this case, the chromosome from which the sequence came from. To map U3 *att* sites, the conserved 5′ LTR ten base pair region (the 5′ *att* site) was used as a query in a simple text editor word search against maize and sorghum sequence, using both the plus and minus strands and with no mismatches allowed. To validate our method, the 3 kb region downstream from the conserved 10 bp sequence was extracted for all matches in maize chromosome 1. Extracted sequences were then used as a query against all Del LTRs extracted from the ten plant genomes (BLASTn, cut-off e-value of 1e^−10^). The figures for the complete and *U3 att* matches were normalized by dividing the number of hits by the length of each chromosome then multiplying by 5×10^6^, so that the final copy number was expressed as the number of copies per five megabases (5 Mb).

### Selective Pressure of Del Lineage among All Genomes

In order to evaluate if the Del elements are under selective constraint, the coding sequences used in the phylogenetic reconstruction were divided according to the groups in the phylogenetic analysis, re-aligned with the ClustalW package v1.81 [23] and manually curated using the amino acid alignment. Codons with alignment gaps and sequences with *indels* longer than five amino acids were excluded from the analysis in order to preserve the reading frames. The largest groups (VII and IX) were split into random subgroups to optimize the computational analyses.

In order to compare codon evolution models to determine selective constraint, three models were tested using the CODEML program from the PAML suite [24]. CODEML performs likelihood ratio tests of hypotheses by evaluation of non-synonymous (dN) and synonymous (dS) distances, and the dN/dS ratio (dN/dS). dN/dS is a signal of the selection at protein level thus, 0≤dN/dS<1 indicates purifying selection, dN/dS = 1 neutral evolution, and dN/dS>1 indicates positive selection. The first model (M0, One Ratio), assumes that all codons across the sequences have the same level of dN/dS. The model M1a (Nearly Neutral) proposes that there two classes of codon, some with 0≤dN/dS<1 and the remainder with dN/dS = 1. Finally, model M2a (Positive Selection) divides codons into three classes: those with 0<dN/dS<1, dN/dS = 1, and dN/dS>1. The fit of model M0 versus M1a or M1a versus M2a is evaluated by a likelihood ratio test comparing twice the difference in log likelihoods with a X^2^ distribution [24]. In M0 versus M1a and M1a versus M2a the degrees of freedom (df) are 1 and 2, respectively.

The codon usage bias was determined by the effective number of codons (*Nc*) value computed by the CodonW program (http://mobyle.pasteur.fr/cgi-bin/portal.py#forms::codonw). *Nc* varies between 21 for maximum codon bias, when only one codon is used per amino acid, and 61 for minimum codon bias, when synonymous codons for each amino acid are used at similar frequencies.

## Results

### Retrieving Del Copies from Ten Genomes

Ten full sequenced angiosperm genomes were selected, from five monocot (*S. italica, S. bicolor, Z. mays, O. sativa* and *B. distachyon*) and five eudicot species (*A. thaliana, M. truncatula, P. trichocarpa, G. max* and *V. vinifera*), representing the two major angiosperm classes. The ten genomes were analyzed using LTR_STRUC [19] to identify LTR retrotransposons based purely on structural criteria. Hence, the present study focuses on LTR retrotransposons with two intact LTRs. 2432 sequences were assigned to the Del lineage using a HMMR profile approach analyzed within a phylogenetic framework (Table S1). The LTR_STRUC program occasionally retrieves sequences where the LTRs have been truncated by the program. For the LTR analysis we excluded these sequences, the final number of Del sequences examined was 2187 (Table S1).

### Phylogenetic Analysis

**Figure 1:**
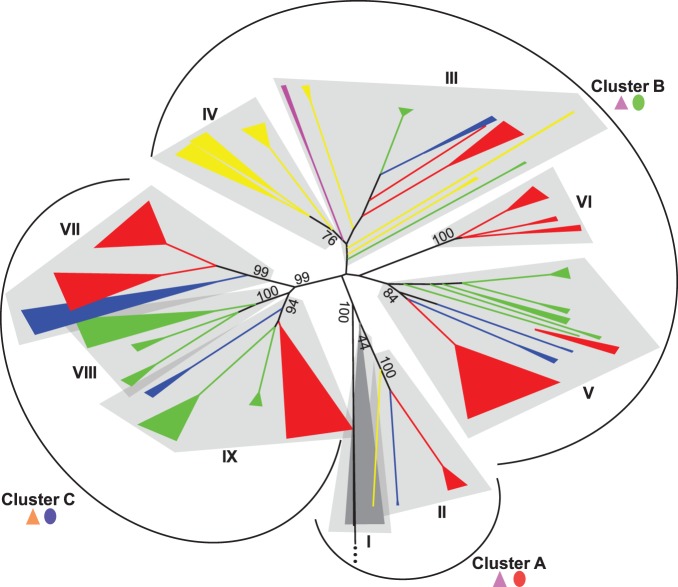
Phylogenetic tree of Del lineage in plants. The neighbor-joining phylogeny was inferred with MEGA5 [21] using the highest-ranked substitution model available (Tamura 3-parameter with gamma distribution of 1.07) and a bootstrap of 100 replicates. The tree is based on a 2389 bp alignment of the RT and RNAseH coding domain, with a total of 2453 sequences (including outgroups). Sequences from the Reina, CRM and Galadriel families [10], [18] were used as outgroups. Groups identified with high bootstrap values are numbered with roman numerals (I to IX). A, B, and C indicate group clusters with shared LTR features. Colors indicate from which genome the sequences came from; grey = eudicots (*A. thaliana, M. truncatula, P. trichocarpa, G. max, V. vinifera),* blue* = S. italica,* green* = S. bicolor,* red* = Z. mays,* yellow* = O. sativa* and pink = *B. distachyon.* Purple triangle = U5 *att* type A; orange triangle = U5 *att* type B; red oval = LTR CpG island pattern type A (no CpG island); green oval = LTR CpG island pattern type B (one CpG island); blue oval = LTR CpG island pattern type C (two CpG islands). Details on *att* types in shown in Figure S2, and on CpG island types are shown in Figure 3. Subgroups are listed in Table 1. Outgroups are shown as a black line with 3 dots.

Del sequences were assigned to groups by phylogenetic analysis based on the RT-RNaseH domains so that we could identify shared features and differences between groups and genomes. Nine main groups were identified irrespective of the plant species and assigned numbers from I–IX (Figure 1). Sequence identity between LTRs is often used to classify LTR-RT lineages into families [1]. All the groups are monophyletic, supported by high bootstrap values (>75), with exception of Group III and Group I, that are monophyletic but have low bootstrap values. Group IV is actually a subgroup derived from Group III, and thus was treated independently because it is monophyletic and supported by a high bootstrap value. Groups IV, VI and VIII are comprised of sequences from a single genome; rice, maize and sorghum, respectively. All the eudicot sequences fell into Group I, while the other eight groups are comprised of exclusively monocot sequences. The monocot Group II is most closely related to the eudicot Group I. Groups VII, VIII and IX fell into a single large group (cluster C), supported by a bootstrap value of 99, in a clade separate from all the other groups. High bootstrap values support the division of these groups into well-defined subgroups, each one with similar LTRs in terms of size, sequence content and features (Table 1).

**Table 1:** Summary of Del retrotransposon features, by group and subgroup.

Group	Subgroup	Total no.	Final nxo.	Genome	CpG island		U5 att type	Average LTR	% nucleotide identity		Bootstraps
		sequences	sequences		✓ = presente ✗ = absent			length in bp	within	within	
					5′	3′		(sd)	5′ CpG island	rest of LTR	
I	a	11	10	At	✗	✗	A	1190.4 (75.3)	−	*	100
	b	17	16	Vv/Pt	✗	✗	A	2187.3 (81.5)	−	*	88
	c	10	6	Gm	✗	✗	A	2397.3 (30.4)	−	93.3	100
	d	23	19	Gm	✗	✗	A	3737.1 (169.8)	−	85.1	100
	e	57	43	Mt	✗	✗	A	2131.4 (34.2)	−	*	100
II	a	1	1	Os	✗	✗	A	2113.0 (−)	−	−	−
	b	3	3	Si	✗	✗	A	2162.7 (2.3)	−	56.1	100
	c	60	53	Zm	✗	✗	A	2381.1 (100.9)	−	52.9	100
III	a	11	8	Bd	✓	✗	A	3251.8 (77.6)	51.0	74.8	100
	b	20	20	Os	✓	✓	A	3875.8 (97.8)	76.4	93.0	100
	c	39	33	Sb	✓	✗	A	2652.9 (138.8)	74.2	92.9	100
	d	20	16	Si	✓	✗	A	2819.0 (150.1)	42.3	80.8	100
	e	1	1	Zm	✓	✗	A	2915.0 (−)	−	−	−
	f	87	83	Zm	✓	✗	A	2823.8 (35.6)	63.4	80.6	100
	g	1	1	Os	✓	✗	A	4235.0 (−)	−	−	87
	h	8	7	Os	✓	✓	A	3861.6 (112.4)	51.9	84.0	100
	i	4	4	Sb	✓	✓	A	3946.8 (18.2)	61.0	89.6	100
IV	a	72	68	Os	✓	✓	A	3094.7 (166.9)	57.3	79.6	100
	b	63	63	Os	✓	✗	A	2953.1 (79.3)	69.9	83.8	100
	c	75	67	Os	✓	✗	A	3152.1 (9.9)	85.7	93.2	100
V	a	51	48	Sb	✓	✓	A	2719.6 (95.4)	43.0	81.1	100
	b	19	14	Sb	✓	✗	A	2846.6 (38.4)	71.5	92.1	100
	c	25	22	Sb	✓	✗	A	2926.0 (51.1)	56.9	78.7	98
	d	10	9	Sb	✓	✓	A	3186.2 (10.4)	87.1	93.3	100
	e	1	1	Sb	✓	✗	A	3292.0 (−)	−	−	100
	f	32	27	Zm	✓	✗	A	3400.9 (122.7)	36.7	77.0	100
	g	3	2	Si	✓	✓	A	2821.0 (62.2)	87.2	85.5	100
	h	17	17	Si	✓	✗	A	2803.0 (77.7)	61.4	90.9	100
	i	337	318	Zm	✓	✗	A	3323.1 (106.3)	29.9	69.9	100
VI	a	70	60	Zm	✓	✗	A	2718.4 (203.6)	47.6	82.2	100
	b	17	14	Zm	✓	✗	A	3571.6 (96.8)	56.8	75.6	100
	c	24	16	Zm	✓	✗	A	3687.2 (79.1)	54.3	81.8	100
VII	a	126	110	Si	✓	✓	B	3165.1 (30.6)	77.6	82.0	98
	b	191	173	Zm	✓	✓	B	4670.5 (34.9)	78.4	86.8	100
	c	287	264	Zm	✓	✓	B	4347.8 (34.2)	72.1	79.8	96
VIII	a	41	35	Sb	✓	✓	B	4498.9 (23.2)	74.7	88.4	100
	b	34	29	Sb	✓	✓	B	4559.1 (61.2)	74.3	87.7	100
	c	147	141	Sb	✓	✓	B	3941.3 (31.0)	62.6	88.7	98
IX	a	209	167	Zm	✓	✓	B	4025.3 (24.3)	46.5	72.9	56
	b	36	36	Sb	✓	✓	B	3935.7 (94.3)	54.5	89.4	100
	c	141	131	Sb	✓	✓	B	4160.3 (100.6)	53.1	89.2	100
	d	31	31	Si	✓	✗	B	4076.7 (41.8)	70.3	83.7	100

Grey shading indicates clusters of groups by LTR feature.

### Identifying Putative Attachment Sites

**Figure 2:**
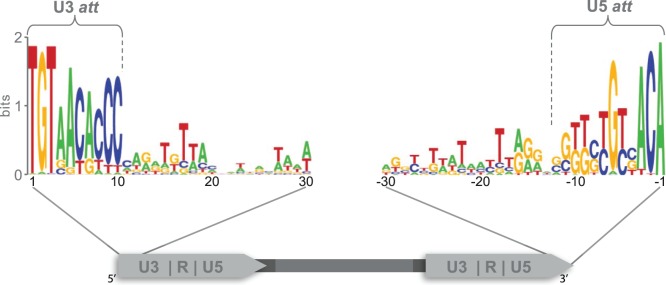
Sequence logos of putative U3 and U5 *att* sites for all Del elements. A schematic representation of an LTR-RT, with the U3, R and U5 regions shown within the LTRs, is at the bottom of the diagram. Sequence logos of the first and last 40 bp of the LTR for all Del elements are shown above. The U5 *att* can be subdivided into two groups (Figure S2), while the U3 *att* is conserved within genomes (Figure S1). Lines show the position of the *att* sites within the LTR. The sequence logo is a graphical representation of a nucleic acid multiple sequence alignment. Each logo consists of stacks of symbols, one stack for each position in the sequence. The overall height of the stack indicates the sequence conservation at that position, while the height of symbols within the stack indicates the relative frequency of each nucleic acid at that position.

Retroviruses and LTR-RTs share several structural features, among the *att* sites. *Att* sites confer specificity to the integration process in retroviruses [12]–[14]. They have been previously described for some LTR-RTs [15], [16], but have not been explored in detail across plant genomes. Two short conserved regions were identified at both ends of the LTR-RTs in the position where retroviral *att* sites are found [12]. The region at the 5′ end of the LTR, in the U3 region, which we propose is the U3 attachment site (U3 *att*), is 10 bp long with 72.2% mean nucleotide identity for all Del sequences (Figure 2). The region at the 3′ end of the LTR, located at the U5 region, which we propose is the U5 attachment site (U5 *att*), is 11 bp long with 61.9% mean nucleotide identity (Figure 2). A comparison of the U3 *att* site for each genome shows that, after the highly conserved dinucleotide (TG/CA), bases 3, 6, 9 and 10 are the most highly conserved in all genomes examined (Figure S1). U5 *att* nucleotides −1, −2, −3 and −6, are conserved in all Del sequences. Two major types of U5 *att*s were identified based on nucleotide differences in positions −8, −9 and −10. Sequences from Groups I–VI in the phylogenetic tree (cluster A and B) have type A U5 *att* with GGG at these positions, while groups VII, VIII and IX (cluster C) have type B U5 *att* with TTC (Figure S2).

The RT-RNaseH domain phylogeny was used to assign Del sequences to groups because phylogenies based on these domains are widely used and have been shown to be robust [4]. A phylogeny based on the integrase domain was also inferred because it is the integrase protein that catalyzes the insertion of reverse-transcribed DNA into the host genome and therefore interacts with the *att* sites [25] [26]. The integrase domain phylogeny showed a similar topology to that of the RT-RNaseH domain phylogeny. The same groups were identified within three large clusters (Figure S3). The number of sequences used for the integrase phylogeny is less than for the RT-RNaseH phylogeny for two reasons. First, some sequences lacked the integrase domain, and second, sequences that were 100% identical were removed.

### Exploring Differences in the LTRs

**Figure 3:**
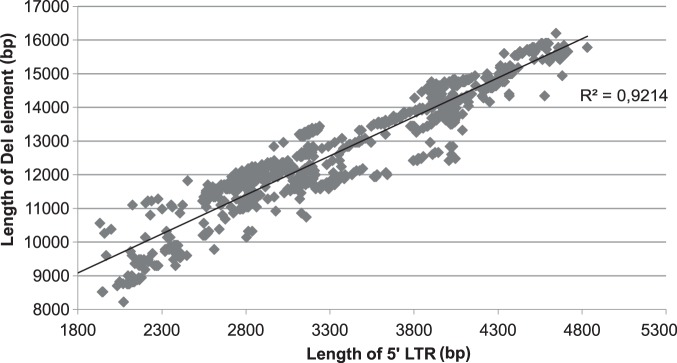
Correlation between LTR length and length of the entire element. The length of the LTR and the complete element were taken from the LTR_STRUC output. R^2^ was calculated using Microsoft Excel. There is a strong positive correlation between the length of LTR and the complete element (R^2^ = 0.92141).

We explored variations in LTR length and correlated this with the presence of one or more CpG islands within the LTR, which is known to include the promoter region of the LTR-RT. CpG islands are important because methylation of CpG sites in the promoter of a gene may inhibit gene expression, this mechanism is also one of the ways in which genomes control TEs. Extensive length variation in LTRs was identified, from 1143 bp in an *Arabidopsis* sequence to 4831 bp in a single sequence from maize (Table 1). We compared the length of the LTR and the length of the internal coding region with the length of the full-length element. We also compared the length of the internal coding region with the length of the full-length element. A strong positive correlation between the length of the LTR and the length of the full-length element was identified (R^2^ = 0.92141) (Figure 3).

**Figure 4:**
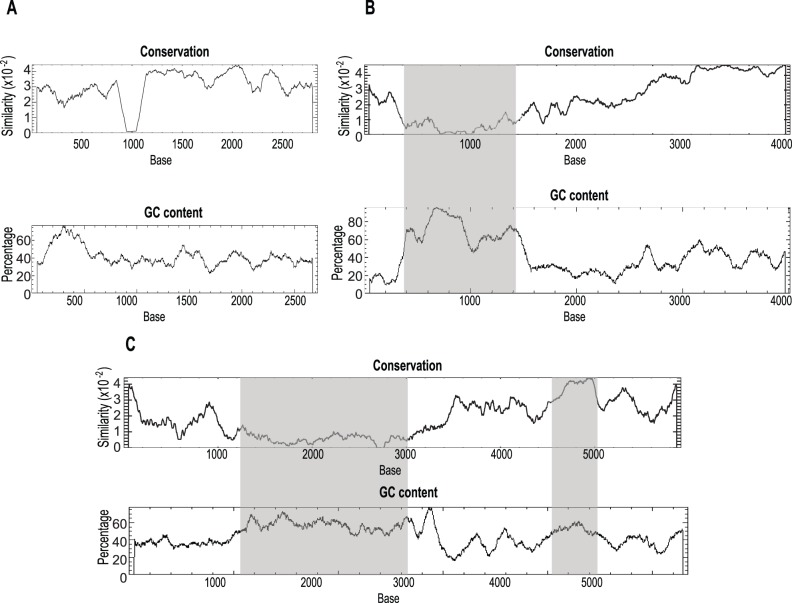
Examples of the three types of patterns of CpG islands identified within LTRs. Graphs show the conservation plot (top graph) and %GC content (bottom graph). CpG islands identified by the CpG plot software indicated by the dark grey (5′CpG island) and light grey (3′ CpG) bars. A is no CpG island identified (example is from subgroup c, group I in Table 1); B is a single CpG island identified in the 5′ half of the LTR (example is from subgroup f, group IV in Table 1) and C is two CpG islands identified, one in the 5′ half and one in the 3′ half of the LTR (example is from sub group a, group IX in Table 1).

**Figure 5:**
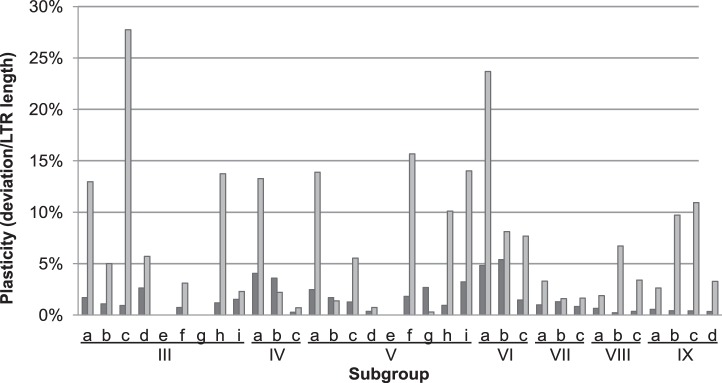
Comparison of size variation of the 5′CpG island compared to the rest of the LTR. Variability in length of 5′ CpG island compared with the rest of the LTR. Groups I and II are not shown because no 5′ CpG island is present. For the remaining groups, the 5′ CpG island is more variable than rest of LTR and is also variable between subgroups. An LTR “plasticity ratio” was calculated for each subgroup identified by phylogenetic analysis. Two regions were compared, the 5′ CpG island identified within the LTR, and the rest of the LTR. For each region, the standard deviation of the length was divided by the average length of that region. This was done for each subgroup. Light grey bars represents the plasticity ratio for the CpG island, dark grey bars the plasticity ratio for the rest of the LTR.

Two putative CpG islands were identified, one at the 5′ and the other at the 3′ end of the LTR (Figure 4). Three different patterns were identified, groups with no CpG islands (cluster A in the phylogenetic tree), those with the 5′ CpG island only (cluster B) and those with both the 5′ and 3′ CpG island (cluster C) (Figure 1 and 4). The island at the 5′ end of the LTR was frequently associated with a region of low sequence conservation and was found in all groups, except groups I and II. The CpG island at the 3′ end of the LTR was frequently associated with a highly conserved region, and was consistently identified in groups VII, VIII and IX (Table 1). The CpG island within the variable region, at the 5′ end of the LTR, was not only variable at the sequence level, but also in length. To quantify this length variation we compared the length of this region with the length of the rest of the LTRs for each subgroup. Interestingly, the CpG island within the 5′ of the LTR showed a higher length variation than the rest of the LTR (Figure 5).

### Uneven Distribution of Del Copies among Chromosomes

**Figure 6:**
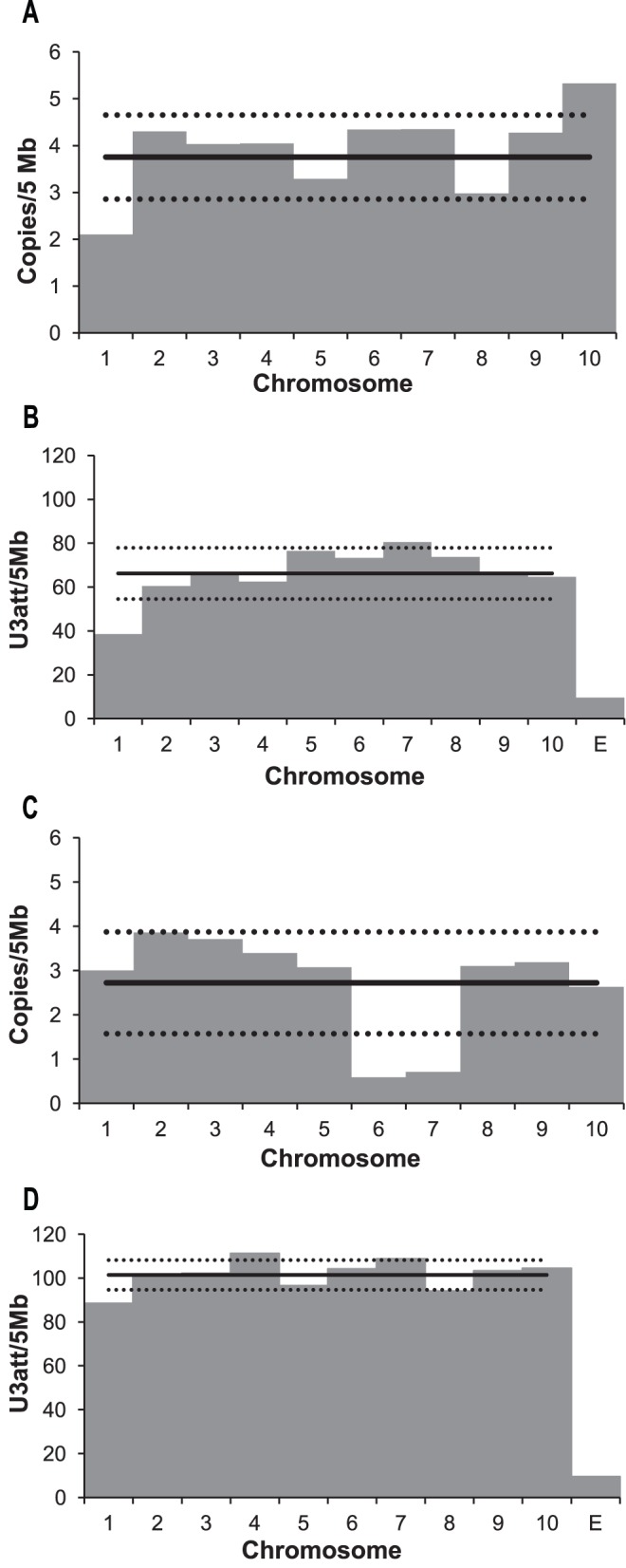
Total number of full-length and U3 *att* matches of Del elements. A and B are sorghum, C and D are maize. The number of full-length copies per 5 Mb was calculated from the LTR_STRUC output (A and C). The number of U3 *att* matches (B and D) was estimated using the number of perfect matches against the U3 *att* consensus sequence: TGTAACACCC. E is the expected frequency of a ten base pair sequence appears by chance, once in each 4^10^ nucleotides. The black horizontal line shows the mean for all chromosomes, the dotted lines show one standard deviation above or below the mean.

**Figure 7:**
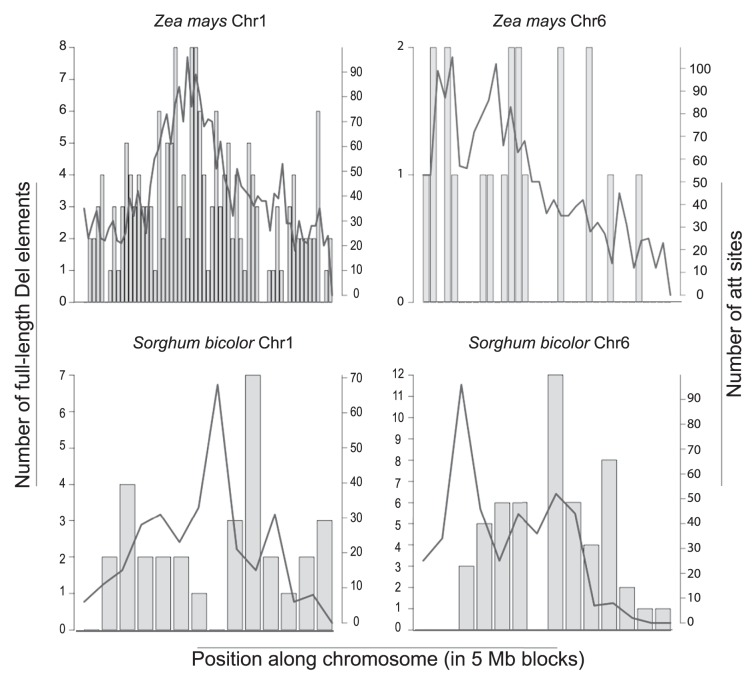
Distribution of U3 *att* and the full-length Del elements along maize and sorghum chromosomes 1 and 6 per 5 Mb. U3 *att* matches are represented by a line. Bars represent the number of full length Del elements located in each 5 Mb.

To test if Del elements are evenly distributed among chromosomes we calculated the number of full-length elements and the most common U3 *att* per 5 Mb for sorghum and maize chromosomes, the genomes with the highest number of copies of Del elements identified (548 and 1315, respectively). A validation of the use of U3 *atts* to identify Del LTRs found that 81.3% and 78.2% of maize and sorghum *att* hits are associated with a Del LTR. Perfect matches with the most common U3 *att* sequence (TGTAACACCC, found in 49.4% of sorghum and 40.7% of maize LTR-RTs found by LTR_SCTRUC) were evenly distributed amongst maize chromosomes (Figure 6). However, full-length elements in both genomes and U3 *att* matches in sorghum were found to be unevenly distributed. This was particularly striking in maize chromosomes 6 and 7, and in sorghum chromosomes 1 and 10. In maize chromosome 6 and 7 less than 1 full-length copy per 5 Mb was identified, while the number of U3 *att* matches was similar to that of the other chromosomes (almost 3 copies per 5 Mb). Sorghum chromosome 1 not only has fewer full-length copies, but also fewer U3 *att* matches than the other chromosomes. Sorghum chromosome 10, in contrast, has more than 5 full-length copies per 5 Mb. The distribution of the 10 bp canonical U3 *att* and the full-length copies (Figure 7) along sorghum and maize chromosomes indicates that the Del elements in general show pericentric accumulation, consistent with the previous findings [27], [28].

### Purifying Selective Pressure of Del Lineage among All Genomes

Previous studies showing that LTR-RTs evolve under purifying selection in plants have been restricted to only one species or to a few copies of an element in more than one species [29]–[31]. In order to gain a wider perspective on the selective constraints shaping LTR-RT evolution, we performed a likelihood ratio test using the same alignment built for the phylogenetic reconstruction. The likelihood ratio test was applied to the nine Del groups separately, since we wished to understand if the group diversification could be related to differential selective constraints. Groups VII and IV contain a large number of sequences and so were subdivided for this analysis due to computational restraints.

The likelihood ratio test assumes three models of coding sequence evolution. The first model, M0, assumes that all codons across the sequences have the same type of selective pressure, purifying selection (0≤dN/dS<1), neutral evolution (dN/dS = 1) or positive selection (dN/dS>1) (See Materials and Methods for details). The second model, M1a, assumes that a proportion of the codons are under purifying selection while the remainders are under neutral evolution. Finally, M2a divides codons into three classes, those with purifying selection, those with a neutral evolution pattern, and the remainder under positive selection. No positive selection was detected, and the likelihood ratio test suggests that the nine groups are evolving under the M1a model (Table 2 and Table S2). The proportion of codons under purifying selection varied from 81% to 98%, and the dN/dS under purifying selection varied from 0.07 to 0.17. No correlation was found when comparing the number of sequences, species, the dN/dS, or the proportion in each group. Low dN/dS values can indicate either high levels of purifying selection (low dN values) on the non-synonymous positions or high values of dS, which could indicate codon usage bias. The mean effective number of codons (Nc) varied from 45.16 to 57.64, suggesting the former alternative, the groups of Del sequences identified in the phylogenetic tree are evolving mainly under purifying selection, with a few codons under neutral evolution.

**Table 2:** Evolutionary models estimated for Del retrotransposon groups calculated using the likelihood ratio test.

	Model	n0^a^	ns^b^	l0^c^	ls^d^	Nc±SD^e^	dN/dS0^f^	p0^g^	dN/dS1^h^	p1^i^	Lnl^j^	2Δl^k^
Group I	M1a	64	63	401	350	47.08±1.65	0.12496	0.81234	1.00000	0.18766	−8697,875106	280,10037**
Group II	M1a	118	114	430	327	45.16±4.22	0.07336	0.97558	1.00000	0.02442	−20432,84542	94,05421**
Group III	M1a	191	184	419	303	51.91±3.05	0.09337	0.95756	1.00000	0.04244	−24090,90373	169,97344**
Group IV	M1a	210	207	398	367	57.64±2.71	0.07208	0.97875	1.00000	0.02125	−22624,46821	104,43849**
Group V	M1a	493	468	557	142	52.21±5.85	0.13739	0.89459	1.00000	0.10541	−34648,36761	961,66957**
Group VI	M1a	111	107	401	348	49.74±1.54	0.10850	0.91622	1.00000	0.08378	−15476,52829	298,27066**
Subgroup VII_1	M1a	604	149	539	206	54.33±2.88	0.08353	0.90928	1.00000	0.09072	−14952,88093	252,14997**
Subgroup VII_2	M1a		148		206		0.09173	0.93315	1.00000	0.06685	−14946,15303	189,97800**
Subgroup VII_3	M1a		149		206		0.08649	0.90713	1.00000	0.09287	−15350,76187	217,61357**
Subgroup VII_4	M1a		148		206		0.09886	0.93927	1.00000	0.06073	−14938,8285	129,69276**
Group VIII	M1a	224	219	424	315	52.05±1.76	0.12089	0.95777	1.00000	0.04223	−23828,23368	165,50066**
Subgroup IX_1	M1a	417	137	462	256		0.08258	0.96316	1.00000	0.03684	−17958,55439	103,59985**
Subgroup IX_2	M1a		136		256	56.36±3.97	0.07854	0.96217	1.00000	0.03783	−16841,5793	92,44688**
Subgroup IX_3	M1a		135		256		0.08074	0.95915	1.00000	0.04085	−16357,5103	208,53267**

Only significant models are shown, for groups with non significant models see Table S2.

a number of sequences in the alignment used to reconstruct the trees.

b number of sequences considered for the likelihood ratio test analysis.

c number of codons in the alignment used to reconstruct the tree.

d number of codons used in the likelihood ratio test analysis.

e mean and standard deviation of the effective number of codons.

f dN/dS estimates assuming a single dN/dS ratio per element.

g Estimated proportion of codons under purifying selection.

h dN/dS estimates assuming neutral evolution.

i Estimated proportion of codons under neutral evolution.

j Log likelihood of model.

k The likelihood ratio statistic (2Δl) is approximated by the X_2_ distribution. Test of M1a vs M0 (line M1a; df = 1) or test M2a vs M1a (line M2a; df = 2).

**: P<0.001.

-: not available.

## Discussion

LTR-RTs are a major component of plant genomes and have been shown to have an impact on genome evolution [32]. In this work, we explore Del elements from 10 plant genomes, to examine LTR diversity and variability in length and sequence within a phylogenetic framework. Del elements have the largest and most highly variable LTRs, and are broadly distributed in all sequenced plant genomes [18]. Our results suggest that the LTRs play an important role both in integration specificity into the host genome and in avoiding host control by methylation. We also propose that they are the major contributors to Del element length variation and that Del sequences are evolving mainly under purifying selection with a few codons under neutral evolution.

### Attachment Sites – Integration Specificity

Attachment (*att*) sites have been previously described in retroviruses, they are characterized by a conserved 8–12 bp region, are found at each end of the retroviral sequence, and are the only viral sequences required in *cis* for recognition by the integration machinery [12], [33]. *In vitro* assays show that in retroviruses the integrase has the ability to interact with the *att* site to mediate integration [14], further, it has also been shown that the integrase assembles independently on the U3 and U5 *att* sites to form a synaptical complex [12], [34]. It has been shown that within the *att* sites only a few nucleotides are essential to confer specificity to the interaction with the integrase protein. These essential nucleotides are called interaction sites [12], [14], [33]. Other studies have demonstrated that single point mutations within the *att* sites reduces or eliminates the capacity of the integrase to recognize the LTRs, hence inhibiting transposition [12], [13]. Although previously described, LTR-RTs *att* sites have not been explored in depth before. A total of 2187 copies with two LTRs were examined for similarly conserved sites; the first 10 bp and last 11 bp of Del elements are highly conserved and independent of the genome in which they are found (Figure 2 and Table 1). The conservation of these bases in more than two thousand sequences supports the idea that they may have a role in the retrotransposon life cycle.

The aligned U3 *att* site sequences share 72.2% identity at the nucleotide level, while the U5 *att* sequences were less conserved, with 61.9% identity. We identified several nucleotides within the U3 and U5 *atts* that were more conserved than others; we suggest therefore these are the most likely interaction site candidates. The U5 *att* sites fell into two groups, type A and type B, explaining the lower percentage identity within the U5 *att* site compared to the U3 *att* site (Figure 2 and Figure S2). Interestingly, the two types of U5 *att* sites are distributed amongst groups throughout the two distinct phylogenetic tree branches, whereas the type B U5 *att* is found only in elements of groups VII−IX (cluster C). The type A U5 *att* site probably represents the ancestral type for the Del lineage since it is shared by all the eudicots and the monocots except for groups VII−IX. The U3 *att* site was more highly conserved amongst all sequences. A second type of U5 *att* has therefore appeared without any changes within the U3 *att* site. The fact that the *att* sites are recognized independently by the integrase suggests that the appearance of this second type of U5 *att* site may be functionally important [12], [34]. The U3 *att* sites are conserved within but not between different genomes, suggesting that these sites may be useful in classifying elements into lineages and also for estimating the number of LTRs present in a genome, as demonstrated here (Figure 6). If the *att* sites are as conserved in retrotransposons as they are in retroviruses, we expect that these sequences could be used to identify autonomous lineages, whose machinery is used by non-autonomous retrotransposons and possibly also by Large Retrotransposon Derivatives (LARDs).

The three clusters identified by the RT-RNaseH phylogeny are also present in the integrase domain phylogeny (Figure S3). Clearly, there are integrase sequence differences associated with the distinct LTR U5 *att* types and GC islands identified. To our knowledge the three dimensional structure of an LTR-RT integrase has not been described. The best studied integrases is those of the retroviruses, particularly that of HIV-1 [35]. The integrase amino-acids that cross-link to the end 3 bases of the HIV LTR are glutamine 62, tyrosine 143, glutamine148, lysine 156 and lysine 159 [36]. Using a HMM-HMM comparison we were able to identify the conceptually translated amino acids in the same relative positions in the Del integrase (data not shown). However, the amino acids were different and had different biochemical properties. The determination of the three dimensional structure of an LTR-RT integrase may allow us to more precisely determine how specificity between the integrase domain and the LTR *att* sites occurs.

### LTR is the Most Variable Region in Terms of Length

Previous study indicates that the variability in length of LTR-RT can be due to variability in the LTR length, most commonly, or due to the variability of the non-coding spacer regions between LTRs and coding regions, as for Tat lineage [10]. To address this issue we compared the length of the LTR with the length of the whole element and the length of the internal coding region, as well as the length of the internal coding region with the whole element. No correlation was found between the length of the internal coding region and the whole element or the LTR. However, there was a significant correlation between the length of the whole element and the length of the LTR. The length of LTR, therefore, is the major contributor to differences in the length of the element, and not expansions or deletions within the coding regions (Figure 3).

### CpG Islands in the LTR

Besides being a key component of retrotransposon integration, the LTRs are also important in expression regulation, as they contain the LTR-RT promoters, enhancers and other regulatory components. The best known mechanism by which genomes maintain LTR-RTs silencing is by methylation of the LTRs [9]. Low complexity CpG rich regions just upstream of the TATA box have been previously described in LTR-RTs in the monocots [37]. These CpG islands are generally found unmethylated in the promoter regions of active genes, hypermethylation of these islands results in an epigenetically silent state. Previous studies have also shown that when a Sp1 motif is present within the CpG island at the 5′ region of the LTR of the Rous sarcoma virus or within the promoter of a gene, host methylation of the promoter is inhibited [38], [39].

We describe three distinct patterns of CpG island distribution amongst examined Del LTR-RTs (Figure 4). The first pattern is no CpG islands within the LTRs. This pattern was predominant in all elements found in cluster A, which includes all eudicot LTR-RT elements and it is the cluster most closely related to the outgroup (with sequences from other Gypsy lineages). The second pattern is LTRs with a single CpG island, commonly found in cluster B. These CpG islands were located at the 5′ end of the LTRs and are associated with regions of low sequence conservation (Figure 4 and Table 1). These CpG islands are more variable in length than the rest of the LTR. As this region is a known target of host genome silencing, we suggest that these 5′ CpG islands may be associated with escaping host control, because of their length variability and low conservation. The third pattern is two CpG islands, the 5′ CpG island described above but also a second CpG island located at the 3′ end of the LTR (Figure 4). Unlike the 5′ CpG islands, 3′ CpG islands are associated with high sequence conservation. This third pattern is present in elements from cluster C. This cluster has another distinctive feature, the unusual U5 type B *att,* and contains sequences only from *S. bicolor, Z. mays* and *S. italica*. The distribution of the three CpG island patterns within the angiosperms suggests that highly variable CpG islands within LTRs is a derived characteristic in the monocots. The possible function of CpG islands in the element’s life cycle remains to be demonstrated.

### Del Retrotransposon Evolution

Using a phylogeny based on RT-RNAseH nucleotide sequences, all full-length Del sequences from the 10 different genomes fell into 9 groups. All 118 eudicot sequences formed a single monophyletic group (Group I). The other 8 groups were composed of Del retrotransposons from monocots, with a total of 2314 sequences (Figure 1). Although larger genomes tend to have more TEs, *G. max* has a genome size of 975 Mb and only 33 complete Del sequences, while *O. sativa* has a genome size of 372 Mb genome and 240 complete Del sequences. Our results suggest that Del elements in grasses, except for *B. distachyon,* have increased copy numbers and diversification compared to those from the eudicot genomes. It is tempting to speculate that these differences could relate to either differences in methylation or sRNA silencing efficiency.

Each LTR feature identified (CpG islands, *att* site, LTR length and plasticity) was characterized by group (Figure 1). Cluster C contains only sequences from *S. italica, S. bicolor and Z. mays,* and forms a monophyletic group supported by a bootstrap value of 99. Cluster C LTRs share some distinguishing features. They all have a unique type of U5 *att* site compared with all other Del sequences and all have two CpG islands (except for subgroup IXd, Table 1). The three species are closely related phylogenetically, therefore, these results suggest the emergence of a subclade within the Del lineage which is exclusive to some grasses. On the ­other hand, no CpG island was identified in sequences from the eudicot group (Group I) or the most closely related monocot group (Group II). These 2 groups are the least successful in terms of copy number and diversity of Del elements.

### Uneven Distribution of Del among Chromosomes

Transposable elements are known to have preferential sites of insertion and are often found in clusters [40]. Unexpectedly, Del copies are unevenly distributed amongst maize and sorghum chromosomes. Two cases are particularly noteworthy. Sorghum chromosome 1 has fewer complete copies and fewer U3 *att* matches (representing complete copies, truncated copies) than other sorghum chromosomes. This may be due to an insertion inhibition or by faster turnover in this chromosome, neither of which have ever been described. A known mechanism of retrotransposon turnover is the recombination between the two LTRs of a single retrotransposon, generating solo LTRs [41]. During the formation of solo LTRs one LTR and the internal coding region of the elements are removed from the genome. Faster turnover in some chromosomes could therefore be mediated by differences in recombination rates among chromosomes, which has been described in mouse [42]. This could explain the low number of both U3 *att* matches and full-length elements on chromosome 1.

On the other hand, maize chromosomes 6 and 7 have fewer complete Del retrotransposons while both have a similar number of Del U3 *att* matches when compared to the other maize chromosomes. This result indicates a larger number of truncated Del copies in these chromosomes. Alternatively, this result could be explained by a higher number of nested elements in these chromosomes, truncating the pre-existing copies. However, it is unclear what causes this phenomenon and what are the mechanisms involved.

### Selective Pressure of Del Lineage among All Genomes

By analyzing 2432 sequences from 10 plant species we have shown that the nine groups identified from the Del GypsyLTR-RT lineage are all evolving under purifying selection combined with a low proportion of codons under neutral evolution. Purifying selection as the main evolutionary force on the LTR-RT reverse transcriptase domain has been previously described, for about 300 Copia LTR-RTs elements from 14 genomes [29], [30] and for all LTR-RT families (Copia and Gypsy) in the rice genome [31]. In addition, in the study of the rice genome, except for one case of positive selection in the gag domain of one family, the authors found that all the coding domains are evolving under purifying selection [31]. In the present work we show that purifying selection is a common feature of plant LTR-RTs rather than a peculiarity of elements in the rice genome. Moreover, in a meticulous analysis we observed the same pattern in all groups from the Del evolutionary lineage.

Although several lines of evidence suggest that TEs evolve under purifying selection, how purifying selection acts on mobilization dynamics is poorly understood. Le Rouzic et al. (2007) argue that the propagation step in the TE life cycle cannot be directly observed. However, with more genomes being completely sequenced and with improved modeling frameworks [43] our understanding of the dynamics and evolutionary history of TE within genomes is increasing [44], [45]. It appears that upon propagation most TE-derived sequences are nonfunctional. Our results showing that there is a higher proportion of U3 *att* matches than full-length Del elements in the sorghum and maize genomes support this hypothesis. However, we were able to identify a significant number of full-length elements that are diversifying, as evidenced by groups VII−IX with novel *att* sites and CpG island; and whose coding sequences are evolving under purifying selection. It is not clear if this diversification and purifying selection are related to the host’s fitness, the element’s evolutionary success or both the host and TEs are taking advantage of the conservation of the coding sequences of these TEs. If the purifying selection identified is related to host fitness, domestication of TEs could be implied. Additionally, the purifying selection could be interpreted as a symbiotic-like state where both TEs and host genome are mutually benefited, where the genome provides maintenance and the protein apparatus while the TEs contribute to genetic variation through recombination and mobilization. Although we prefer the second hypothesis, which the purifying selection observed is related to TE success, we are unable to explain how equilibrium between replication and selection on the coding sequences versus turnover is maintained. Further interdisciplinary studies including genomic, ecological and population genetic approaches should provide models to explain how purifying selection shapes the evolution of TEs.

## Conclusions

We were able to identify two types of patterns, the first with features common to the entire Del lineage, and the second with features particular to a branch of the phylogenetic tree. The uneven distribution of Del copies in maize and sorghum chromosomes is characteristic of the whole Del lineage, and suggests dissimilar evolutionary histories of TEs on different chromosomes. Another shared characteristic is the variability of LTR length compared to the rest of the retroelement. The purifying selective pressure on the reverse transcriptase domain is common to all the Del groups we examined, suggesting selective pressure on the transposition process instead of the host genome. The alternative is highly unlikely, that coding sequence conservation of the majority of copies from all groups studied is important to host fitness.

This is the first time that the sequence conservation of *att* sites in LTR-RTs has been explored in detail. The *att* sites were found in all Del groups, with two types of U5 *att* sites identified, one of each falling within the two major branches of the tree. It would appear therefore that Del elements are transposed only by integrases of the same lineage. The appearance of a new type of U5 *att* indicates the emergence of a new clade within the Del lineage, with groups VII−IX. On the other hand, the fact that the U3 *att* is highly conserved among all studied genomes corroborates current evidence that this is a fundamental region for integration specificity.

The high sequence and length variation of the 5′ CpG island in the LTR of Del lineage may be associated with methylation and transcriptional silencing, suggesting a way to increase expression or even a form of host silencing avoidance by presenting a ‘moving target’. LTR variability (e.g. variation in CpG island presence, U5 *att* and size) is concomitant with minor changes in the coding regions, as evidenced by the phylogenetic analysis, since each cluster in the tree is composed of elements with different LTR features. The unique features of the Groups within cluster C indicate that this is a new sub-lineage emerging in the monocots. Is the LTR the trigger for the diversification of these retrotransposons? Is this type of process also found in other retrotransposon lineages?

## Supporting Information

Figure S1 **U3 att sequence logos by genome.** The putative U3 att sequence is conserved within genomes. Zm = Z. mays, Sb = S. bicolor, Si = S. italica, Os = O. sativa, Vv = V. vinifera; Mt = M. truncatula; Gm = G. max; Bd = B. distachyon, At = A. thaliana. P. trichocarpa is not included because there is only one sequence. Blue bars indicate highly conserved bases. Please see the legend of Figure5 for a description of a sequence logo.
(PDF)

Figure S2 **U5** ***att*** **type A and B sequence logos.** Two types of putative U5 *att* types were identified, A and B. Type A was found in groups I–VI, while type B was found only in groups VII to IX. Please see the legend of Figure2 for a description of a sequence logo.
(PDF)

Figure S3 **Phylogenetic tree of Del lineage based on integrase domain.** The neighbor-joining phylogeny was inferred with MEGA5 [21] using the highest-ranked substitution model available (Tamura 3-parameter with gamma distribution of 0.8) and a bootstrap of 100 replicates. The tree is based on a1140 bp alignment of the integrase coding domain, with a total of 2358 sequences (including outgroups). Sequences from the Reina, CRM and Galadriel families [10], [18] were used as outgroups.
(PDF)

Table S1 **Total number of Del elements identified in each genome and the number of elements used in LTR analyses.**
(PDF)

Table S2 **Likelihood ratio test for estimating selective constraints in the groups of Del retrotransposons. Non-significant models.**
(PDF)
