# Supplementary figures and images for: Virus-Like Attachment Sites and Plastic CpG Islands: Landmarks of Diversity in Plant Del Retrotransposons
Source: PLoS One. 2014 May 21;9(5):e97099. doi: 10.1371/journal.pone.0097099 (PMC4029996; doi:10.1371/journal.pone.0097099)

Additional file 2 – Figure S1

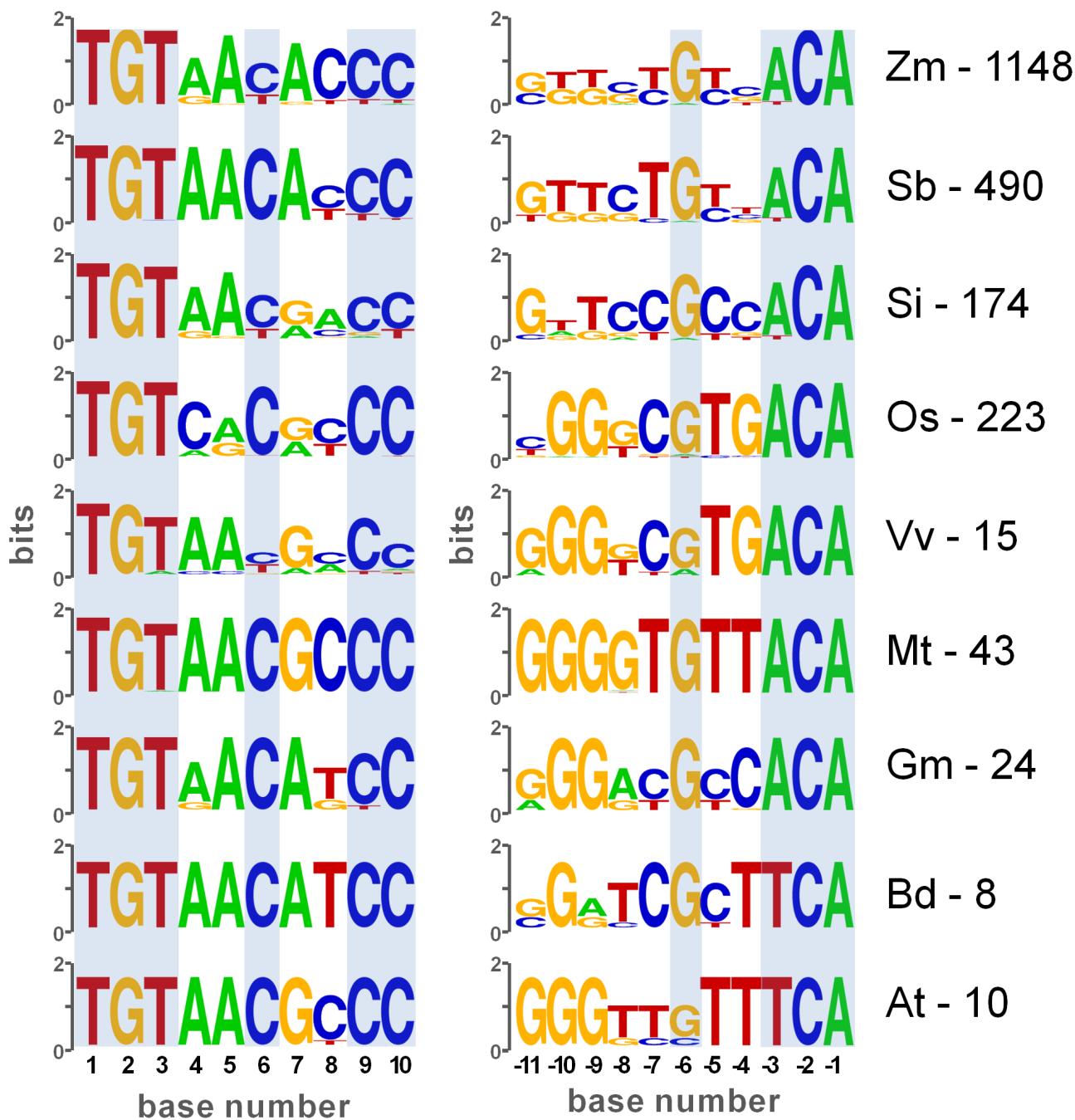

Supplement: Figure S1 — U3 att sequence logos by genome. The putative U3 att sequence is conserved within genomes. Zm = Z. mays, Sb = S. bicolor, Si = S. italica, Os = O. sativa, Vv = V. vinifera; Mt = M. truncatula; Gm = G. max; Bd = B. distachyon, At = A. thaliana. P. trichocarpa is not included because there is only one sequence. Blue bars indicate highly conserved bases. Please see the legend of Figure5 for a description of a sequence logo. (PDF) [file pone.0097099.s001.pdf]

Additional file 3 – Figure S2

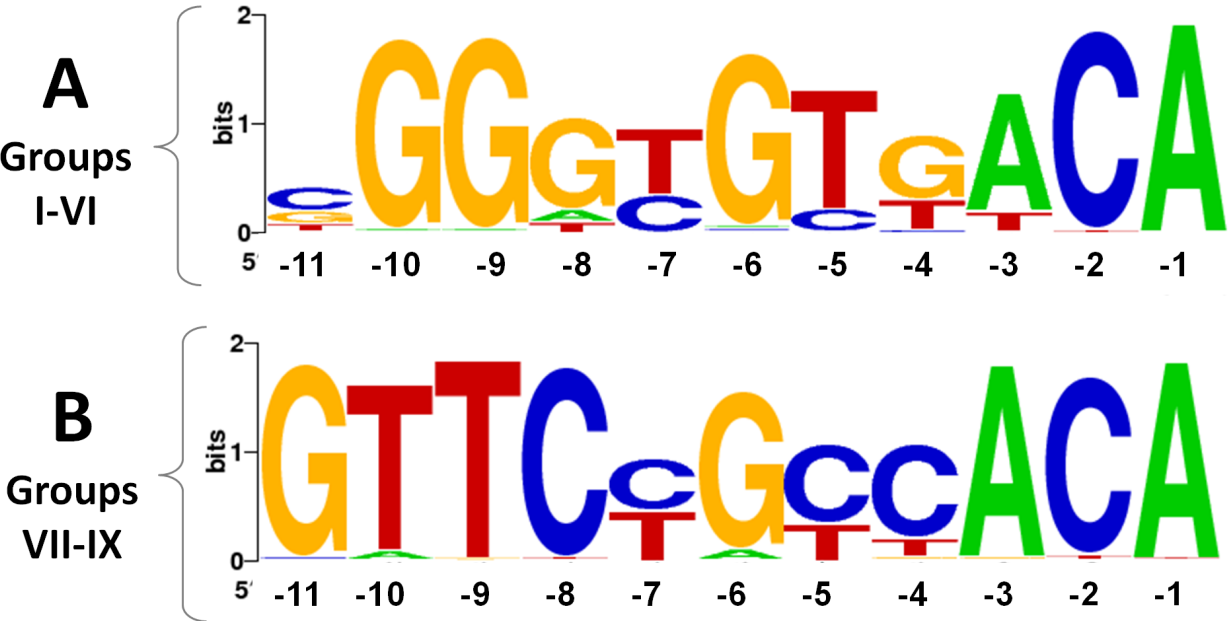

Supplement: Figure S2 — U5 att type A and B sequence logos. Two types of putative U5 att types were identified, A and B. Type A was found in groups I–VI, while type B was found only in groups VII to IX. Please see the legend of Figure2 for a description of a sequence logo. (PDF) [file pone.0097099.s002.pdf]

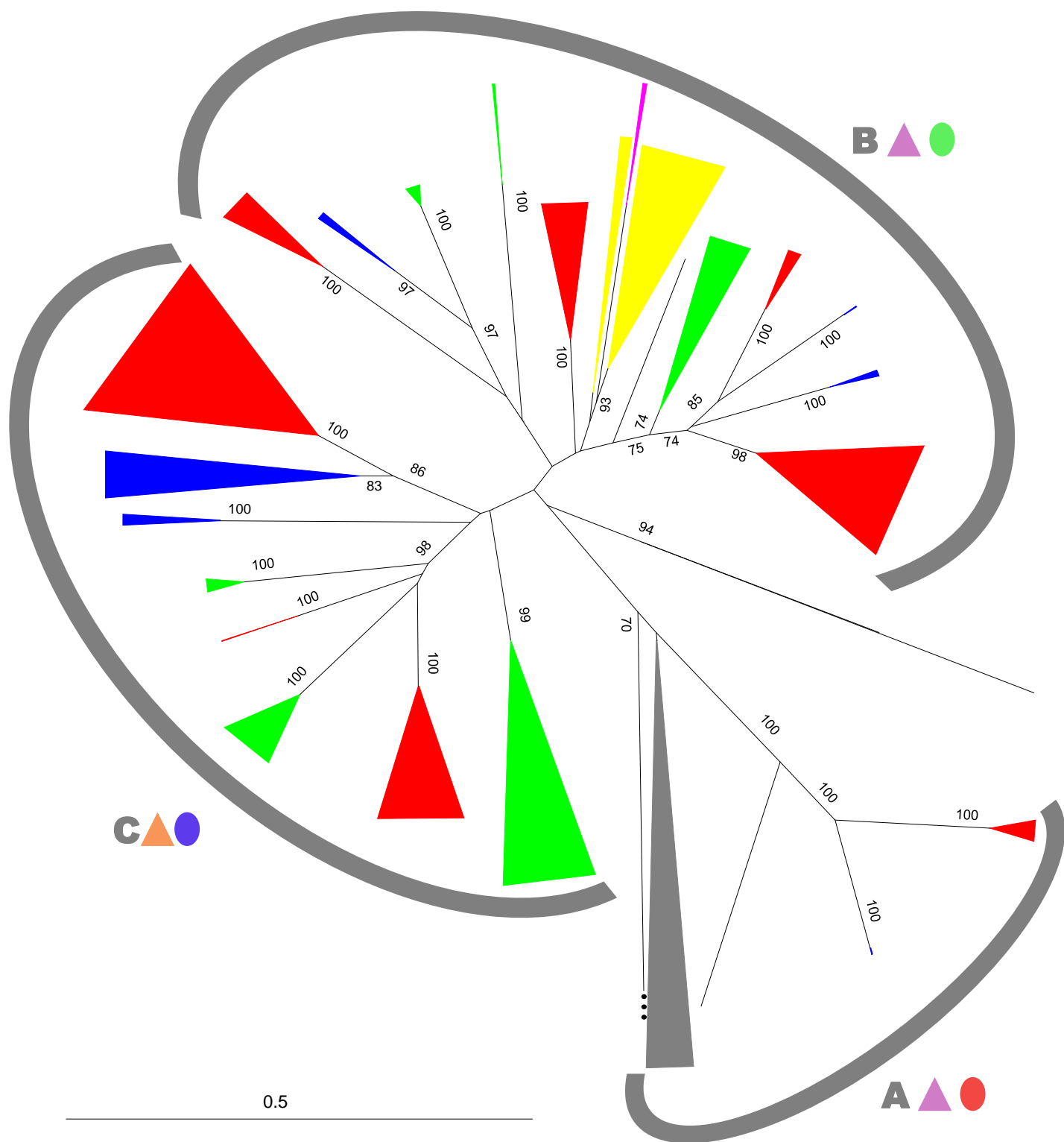

Supplement: Figure S3 — Phylogenetic tree of Del lineage based on integrase domain. The neighbor-joining phylogeny was inferred with MEGA5 [21] using the highest-ranked substitution model available (Tamura 3-parameter with gamma distribution of 0.8) and a bootstrap of 100 replicates. The tree is based on a1140 bp alignment of the integrase coding domain, with a total of 2358 sequences (including outgroups). Sequences from the Reina, CRM and Galadriel families [10], [18] were used as outgroups. (PDF) [file pone.0097099.s003.pdf]
