# Supplementary material for: Virus-Like Attachment Sites and Plastic CpG Islands: Landmarks of Diversity in Plant Del Retrotransposons
Source: PLoS One. 2014 May 21;9(5):e97099. doi: 10.1371/journal.pone.0097099 (PMC4029996; doi:10.1371/journal.pone.0097099)
Supplement: Table S1 — Total number of Del elements identified in each genome and the number of elements used in LTR analyses. (PDF) [file pone.0097099.s004.pdf]

Additional file 1 - Table S1 Total number of Del elements identified in each genome and the number of elements used in LTR analyses.

Genome	nº sequences used in phylogenetic analysis	nº sequences used in the LTR analyses
Eudicots		
Arabidopsis thaliana	11	10
Medicago truncatula	57	43
Populus trichocarpa	1	1
Vitis vinifera	16	15
Glicine max	33	25
Monocots		
Brachypodium distachyon	11	8
Oryza sativa	240	227
Setaria italica	200	179
Sorghum bicolor	548	503
Zea mays	1315	1176
Total	2432	2187
