# Supplementary material for: Virus-Like Attachment Sites and Plastic CpG Islands: Landmarks of Diversity in Plant Del Retrotransposons
Source: PLoS One. 2014 May 21;9(5):e97099. doi: 10.1371/journal.pone.0097099 (PMC4029996; doi:10.1371/journal.pone.0097099)
Supplement: Table S2 — Likelihood ratio test for estimating selective constraints in the groups of Del retrotransposons. Non-significant models. (PDF) [file pone.0097099.s005.pdf]

Additional file 4 - Table S2 Likelihood ratio test for estimating selective constraints in the groups of Del retrotransposons. Non-significant models.

	Model	ω0[a]	p0[b]	ω1[c]	p1[d]	ω2[e]	p2[f]	Lnl[g]	2Δl[h]
Group I	M0	0.22484	1.00000	-	-	-	-	-8837,925291	-
	M2a	0.12496	0.81234	1.00000	0.09764	1.00000	0.09002	-8697,875106	0.00000
Group II	M0	0.07603	1.00000	-	-	-	-	-20479,87253	-
	M2a	0.07336	0.97558	0.00883	1.00000	0.01560	1.00000	-20432,84542	0.00000
Group III	M0	0.10171	1.00000	-	-	-	-	-24175,89045	-
	M2a	0.09337	0.95752	1.00000	0.01775	1.00000	0.02473	-24090,90373	0.00001
Group IV	M0	0.07836	1.00000	-	-	-	-	-22676,68746	-
	M2a	0.07208	0.97873	1.00000	0.01368	1.00000	0.00758	-22624,46822	0.00002
Group V	M0	0.17030	1.00000	-	-	-	-	-35129,2024	-
	M2a	0.13739	0.89454	1.00000	0.07272	1.00000	0.03274	-34646,57439	3,58643
Group VI	M0	0.14802	1.00000	-	-	-	-	-15625,66362	-
	M2a	0.10851	0.91621	1.00000	0.05746	1.00000	0.02633	-15476,52829	0,00000
Subgroup VII_1	M0	0.11217	1.00000	-	-	-	-	-15078,95592	-
	M2a	0.08353	0.90928	1.00000	0.09072	3.84747	0.00000	-14952,88094	0,00001
Subgroup VII_2	M0	0.11300	1.00000	-	-	-	-	-15041,14203	-
	M2a	0.09174	0.93314	1.00000	0.06686	21.32788	0.00000	-14946,15313	0,00019
Subgroup VII_3	M0	0.11310	1.00000	-	-	-	-	-15459,56866	-
	M2a	0.08650	0.90718	1.00000	0.09282	3.50499	0.00000	-15350,68401	0,15571
Subgroup VII_4	M0	0.11637	1.00000	-	-	-	-	-15003,67488	-
	M2a	0.09886	0.93927	1.00000	0.06073	14.01918	0.00000	-14938,82855	0.00010
Group VIII	M0	0.13443	1.00000	-	-	-	-	-23910,98402	-
	M2a	0.12089	0.95776	1.00000	0.01817	1.00000	0.02407	-23828,23368	0.00000
Subgroup IX_1	M0	0.09164	1.00000	-	-	-	-	-18010,35432	-
	M2a	0.08258	0.96316	1.00000	0.03684	43.23232	0.00000	-17958,55439	0.00000
Subgroup IX_2	M0	0.08813	1.00000	-	-	-	-	-16887,80275	-
	M2a	0.07854	0.96217	1.00000	0.03783	18.21582	0.00000	-16841,5793	0.00000
Subgroup IX_3	M0	0.09633	1.00000	-	-	-	-	-16461,77664	-
	M2a	0.08074	0.95915	1.00000	0.04085	18.60650	0.00000	-16357,5103	0.00000
